# Association between breast cancer and thyroid cancer: A study based on 13 978 patients with breast cancer

**DOI:** 10.1002/cam4.1856

**Published:** 2018-11-27

**Authors:** Nai‐si Huang, Xing‐xing Chen, Wen‐jun Wei, Miao Mo, Jia‐ying Chen, Ben Ma, Shu‐wen Yang, Wei‐bo Xu, Jiong Wu, Qing‐hai Ji, Xiao‐mao Guo, Guang‐yu Liu, Zhi‐min Shao, Yu Wang

**Affiliations:** ^1^ Department of Head and Neck Surgery Fudan University Shanghai Cancer Center Shanghai China; ^2^ Department of Oncology, Shanghai Medical College Fudan University Shanghai China; ^3^ Department of Radiation Oncology Fudan University Shanghai Cancer Center Shanghai China; ^4^ Department of Clinical Statistics Fudan University Shanghai Cancer Center Shanghai China; ^5^ Department of Breast Surgery Fudan University Shanghai Cancer Center Shanghai China

**Keywords:** breast cancer, prognosis, second primary malignancy, synchronous cancer, thyroid cancer

## Abstract

**Background:**

Thyroid cancer (TC) is one of the most commonly seen secondary malignancy in breast cancer (BC) survivors.

**Materials and methods:**

A retrospective study was conducted in BC patients in our center from 1999 to 2013. Patients were divided into BC‐TC group and BC‐alone group.

**Results:**

In total, 13 978 BC patients were identified, among whom 247 (1.8%) had TC. The standardized incidence ratio (SIR) of TC was 4.48 compared with Chinese females, and up to 98.0% of cases were thyroid papillary carcinomas. A family history of malignancy was the only independent risk factor (odds ratio = 1.457, *P* = 0.025) for development of TC in patients with BC. We also identified inferior survival in patients with synchronous versus metachronous BC‐TC (*P* = 0.016). Synchronous BC‐TC (risk ratio = 5.597, *P* = 0.018) was an independent prognostic factor for inferior RFS.

**Conclusions:**

We observed high co‐occurrence of TC in patients with BC. There might be different mechanisms behind synchronous and metachronous BC‐TC.

## 1 INTRODUCTION

Breast cancer (BC) is the most common type of cancer and the leading cause of cancer‐related death in women worldwide. In China, the incidence of BC has increased consistently over the years. Estimates showed that 278 800 women were diagnosed with BC and 64 600 women died of this disease in 2013, accounting for 17.07% of all cancers and 7.85% of all cancer deaths in women.^1^ Improvements in diagnostic approaches, early detection of cancer, and advances in treatment have led to improved survival rates in patients with BC.^2^ A considerable number of these long‐term survivors may therefore have an elevated risk of developing a second primary malignancy (SPM).

Thyroid cancer (TC) is the most prevalent endocrine malignancy among women. Epidemiologic studies have indicated that patients with BC have a higher risk of developing TC as an SPM, and vice versa, than would be expected in the general population; and the risk of BC following TC is increased by 21% to 89% and that of TC following BC by 31% to 73%.^2^, ^3^ Therefore, these cancers might share some etiologies in common, including hormonal factors, a genetic predisposition, and environment‐ and therapy‐related factors. However, the elevated incidence of co‐occurrence of TC and BC might be caused by detection bias due to more careful follow‐up after diagnosis of the primary tumor.^4^, ^6^, ^7^ The relationship between BC and TC and the exact mechanism behind their possible association remains elusive.

Here, we performed a retrospective study of 13 978 patients with BC to investigate the incidence of and risk factors for TC and determine whether the occurrence of TC affects the prognosis of BC. An improved understanding of the clinical correlation between TC and BC could help in posttreatment management of these patients.

## 2 MATERIALS AND METHODS

### 2.1 Patients

We conducted a retrospective study of patients with BC who underwent surgery from January 1999 to December 2013 at Fudan University, Shanghai Cancer Center (FUSCC). The following exclusion criteria were applied: (a) metastatic or recurrent BC upon first diagnosis, (b) a lack of follow‐up information, and (c) breast sarcoma, lymphoma, melanoma, or other cancers besides breast epithelial BC.

All patients were divided into two groups according to whether they had surgically proven TC: those with both BC and TC (BC‐TC group) and those with only BC (BC‐alone group). Information regarding thyroid surgery was acquired either by reviewing the disease history in the admission note or by following up patients after breast surgery.

### 2.2 BC and TC features

The BC characteristics included the year of treatment, pathology, grade, tumor size, lymph node status, lymphovascular invasion (LVI), estrogen receptor (ER) status, progesterone receptor (PR) status, human epidermal growth factor receptor 2 (HER2) status, and Ki67 status. ER and PR positivity were defined as tumor cell positivity of >1%, and HER2 positivity was defined as HER2 (+++) on immunohistochemistry or overexpression on fluorescence in situ hybridization. The median Ki67 value was 20% and was therefore adopted as the cutoff value. HER2 and Ki67 were not routinely tested in our institute until 2009. The BC subtypes were categorized based on the 2015 St. Gallen consensus. The TC information included the pathologic subtype, tumor size, lymph node status, and year of treatment. Pathologic assessments were performed based on the final paraffin‐embedded section postoperatively.

Regarding the timing of diagnoses, TC diagnosed within 1 year before or after BC was defined as synchronous malignancy (B = T), TC diagnosed >1 year after BC was considered a metachronous second primary TC (B‐T), and TC diagnosed 1 year before BC was categorized in the T‐B group.

### 2.3 Follow‐up

The follow‐up data for the patient cohort were acquired from the Department of Clinical Statistics of FUSCC, and the last follow‐up was performed on 31 March 2017. Recurrence of BC was defined as progression in the ipsilateral breast, skin, muscles of the chest wall, axillary or supraclavicular lymph nodes, or other organs. Recurrence‐free survival (RFS) was calculated from the date of surgery to the date of clinical relapse. The time interval from TC to BC was also recorded. The present study was approved by the Ethics Committee of FUSCC.

### 2.4 Statistics

The standardized incidence ratio (SIR) of TC in patients with BC was calculated by comparing the number of patients at risk of subsequent TC and the number of cases that would be expected in the general Chinese population. The confidence interval (CI) was calculated by bootstrap sampling. The baseline characteristics of patients in the BC‐TC and BC‐alone groups were compared using Pearson's *χ* ^2^ test. A logistic regression model was used to identify variables associated with TC in patients with BC.

The 5‐year RFS was estimated using the Kaplan‐Meier method, and differences were assessed with the log‐rank test. Univariate and multivariate analyses for RFS were conducted using Cox regression models. Two‐tailed *P* values were adopted, and *P* < 0.05 was considered statistically significant. All analyses were performed using SPSS version 24.0 (IBM Corp., Armonk, NY, USA).

## 3 RESULTS

### 3.1 Incidence of thyroid disease in patients with BC

We identified a total of 13 978 patients with BC who qualified for analysis, 247 (1.8%) of whom were diagnosed with TC (BC‐TC). The mean follow‐up time was 64.5 ± 32.3 months. The mean time interval between BC surgery and TC surgery was 1.13 ± 4.97 years. Specifically, 43 (17.4%) patients had TC >1 year prior to BC (T‐B), 74 (30.0%) had synchronous BC and TC (B = T), and 130 (62.6%) were diagnosed with TC at least 1 year after BC (B‐T).

Based on the cancer incidence in China in 2013, the incidence of TC in both females and males was 7.37/10^5^; that in females was 11.24/10^5^ in the general population and 19.98/10^5^ in areas of high urbanization.^1^ In our cohort of patients with BC, the SIR for second primary TC was 6.92 (95% CI, 6.03‐8.29) for both females and males. The SIR for TC was 4.48 (95% CI, 3.95‐5.44) for females in the general population and 2.52 (95% CI, 2.22‐3.06) compared with the incidence of TC in areas of high urbanization.

Among 247 cases of BC‐TC, 203 had specific pathologic information: 200 were thyroid papillary carcinomas, one was a follicular carcinoma, one was a medullary carcinoma, and one was a combination of both papillary and follicular carcinoma. Among 181 cases of known tumor size, 124 (68.5%) were microcarcinomas. Of 202 patients with a known lymph node status, 137 (67.8%) had no lymph node metastasis, 48 (23.8%) had central neck lymph node metastasis, and 17 (8.4%) had lateral neck lymph node metastasis. Of 185 patients with a known number of thyroid cancer lesions, 53 (21.5%) had multifocal lesions.

### 3.2 Clinicopathological characteristics of BC in patients with TC

The clinicopathological characteristics of patients with BC are shown in Table 1. Patients with BC‐TC were less likely to be menopausal (*P* = 0.004) and more likely to have a positive family history of malignancy (any kind of malignant tumors, including BC, TC, *P* = 0.004) than patients in the control group. In terms of BC features, patients with BC‐TC had a smaller invasive tumor size (*P* = 0.023) and a lower rate of high Ki67 expression (*P* = 0.001) than patients in the BC‐alone group. However, no difference was detected in tumor grade, ER status, PR status, HER2 status, or breast cancer subtype.

**Table 1:** Clinicopathological characteristics of patients with breast cancer and thyroid cancer versus patients with breast cancer alone

Variables	Total	BC‐TC N = 247	BC alone N = 13 731	*P*‐value
Sex				
Female	13 922 (99.6%)	246 (99.6%)	13 676 (99.6%)	0.992
Male	56 (0.4%)	1 (0.4%)	55 (0.4%)	
Age				
≤50	6931 (49.6%)	137 (55.5%)	6794 (49.5%)	0.062
>50	7047 (50.4%)	110 (44.5%)	6937 (50.5%)	
Menopause				
No	5460 (39.1%)	120 (48.6%)	5340 (38.9%)	0.004
Yes	6563 (47.0%)	104 (42.1%)	6459 (47.0%)	
Unknown	1955 (14.0%)	23 (9.3%)	1932 (14.1%)	
Family history of malignancy				
No	9967 (71.3%)	153 (61.9%)	9814 (71.5%)	0.004
Yes	3845 (27.5%)	89 (36.0%)	3756 (27.4%)	
Unknown	166 (1.2%)	5 (2.0%)	161 (1.2%)	
Pathology				
Invasive	12 125 (86.7%)	209 (84.6%)	11 916 (86.8%)	0.218
In situ	1772 (12.8%)	38 (15.4%)	1734 (12.6%)	
Unknown	81 (0.6%)	0 (0.0%)	81 (0.6%)	
Tumor size^a^				
≤2 cm	7514 (53.8%)	154 (62.3%)	7360 (53.6%)	0.023
>2 cm	5158 (36.9%)	73 (29.6%)	5085 (37.0%)	
Unknown	1306 (9.3%)	20 (8.1%)	1286 (9.4%)	
Grade				
I‐II	6642 (47.5%)	129 (52.2%)	6513 (47.4%)	0.243
III	3190 (22.8%)	47 (19.0%)	3143 (22.9%)	
Unknown	4146 (29.7%)	71 (28.7%)	4075 (29.7%)	
pN				
pN0	8806 (63.0%)	169 (68.4%)	8637 (62.9%)	0.141
pN1	2981 (21.3%)	49 (19.8%)	2932 (21.4%)	
pN2‐3	2191 (15.7%)	29 (11.7%)	2162 (15.7%)	
LVI				
Yes	3219 (23.0%)	44 (17.8%)	3175 (23.1%)	0.145
No	6705 (48.0%)	127 (51.4%)	6578 (47.9%)	
Unknown	4054 (29.0%)	76 (30.8%)	3978 (29.0%)	
ER				
Positive	9666 (69.2%)	174 (70.4%)	9492 (69.1%)	0.764
Negative	3738 (26.8%)	65 (26.3%)	3673 (26.7%)	
Unknown	574 (4.1%)	8 (3.2%)	566 (4.1%)	
PR				
Positive	8925 (63.9%)	161 (65.2%)	8764 (63.8%)	0.583
Negative	4477 (32.0%)	79 (32.0%)	4398 (32.0%)	
Unknown	576 (4.1%)	7 (2.8%)	569 (4.1%)	
HER2^b^				
Positive	2661 (21.9%)	48 (23.0%)	2613 (21.9%)	0.582
Negative	8155 (67.3%)	143 (68.4%)	8012 (67.2%)	
Unknown	1309 (10.8%)	18 (8.6%)	1291 (10.8%)	
Ki67				
<20%	3313 (23.7%)	79 (32.0%)	3234 (23.6%)	0.001
≥20%	4051 (29.0%)	78 (31.6%)	3973 (28.9%)	
Unknown	6614 (47.3%)	90 (36.4%)	6524 (47.5%)	
Subtype^b^				
Luminal A	1540 (12.7%)	36 (17.2%)	1504 (12.6%)	0.232
Luminal B	6046 (49.9%)	97 (46.4%)	5949 (49.9%)	
HER2‐enriched	1059 (8.7%)	20 (9.6%)	1039 (8.7%)	
Triple‐negative	1480 (12.2%)	28 (13.4%)	1452 (12.2%)	
Unknown	2000 (16.5%)	28 (13.4%)	1972 (16.5%)	

BC, breast cancer; ER, estrogen receptor; HER2, human epidermal receptor 2; LVI, lymphovascular invasion; pN, pathological N stage; PR, progesterone receptor.

a Size of invasive disease on final pathology.

b Only HER2 status in invasive disease was analyzed.

Among 202 patients with known TC lymph node metastasis, the clinicopathological characteristics were not significantly different between the groups, suggesting that the BC features do not have an impact on lymph node metastasis in patients with TC. Similarly, TC multifocality was independent of BC features. However, high patient age (*P* = 0.022) and a postmenopausal status (*P* = 0.039) were associated with a TC size of >1 cm. Finally, no differences regarding breast cancer clinical‐pathological features were found among the TC‐BC, TC=BC, and BC‐TC groups (Tables S1‐S4).

**Figure 1:**
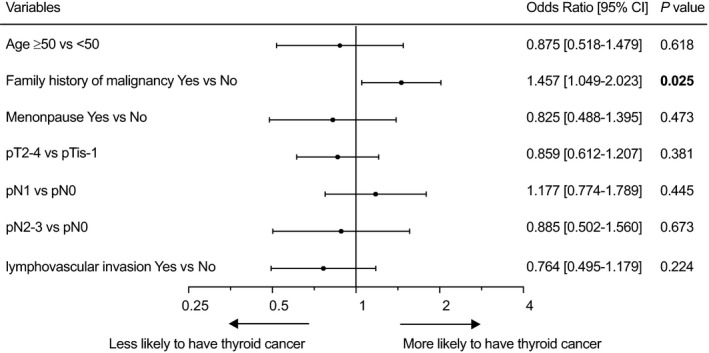
Multivariate logistic regression demonstrated that a family history of malignancy was the only predictor of TC in the cohort of patients with BC

In the univariate logistic regression analysis, we explored the possible TC‐related variants in patients with BC. Generally speaking, premenopausal (odds ratio [OR] = 0.717; 95% CI: 0.550‐0.934; *P* = 0.014) young patients with less aggressive BC were more likely to have TC (Table S5). Patients with a larger tumor size (OR = 0.686; 95% CI: 0.518‐0.909; *P* = 0.009) and more than four lymph node metastases (OR = 0.686; 95% CI: 0.461‐1.019; *P* = 0.062) were less likely to have TC. Moreover, patients with a positive family history of malignancy were more likely to have TC (OR = 1.520; 95% CI: 1.167‐1.979; *P* = 0.002). Variants with *P* < 0.10 were included in the multivariate regression model, revealing that only a family history of malignancy was an independent predictor of TC in patients with BC (OR = 1.457; 95% CI: 1.049‐2.023; *P* = 0.025; Figure 1).

### 3.3 Clinical prognosis of patients with BC diagnosed with TC

The mean follow‐up time in the BC‐TC and BC‐alone groups were 64.31 ± 0.26 and 67.97 ± 1.86 months, respectively. In the survival analysis, patients in the BC‐TC group showed superior RFS over patients in the BC‐alone group (5‐year RFS, 95.1% and 88.9%, respectively; *P* = 0.004; Figure S1). Nevertheless, when the analysis was limited to only patients with lymph node‐negative BC, there were no differences between the groups (5‐year RFS, 96.6% and 94.3%, respectively; *P* = 0.218; Figure S2). Multivariate Cox regression analysis showed that ER negativity (*P* < 0.001), a larger tumor size (*P* < 0.001), lymph node metastasis (*P* < 0.001), and young age at diagnosis (*P* = 0.006) were associated with unfavorable RFS in patients with BC, while the co‐occurrence of TC was not (*P* = 0.437).

**Figure 2:**
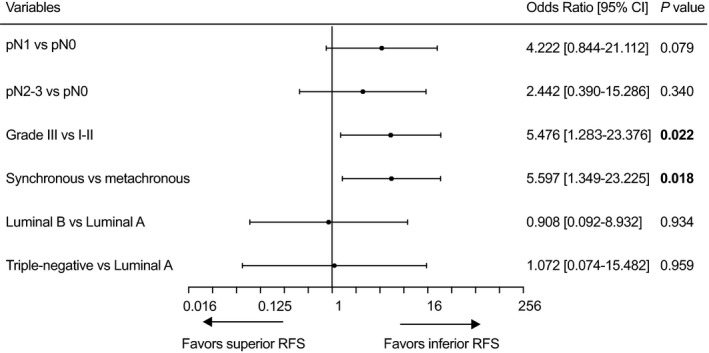
Multivariate Cox regression demonstrated that a high BC grade and synchronous TC were negative predictors of recurrence‐free survival in the BC‐TC group

Finally, we limited our cohort to patients with BC‐TC, among whom 74 had synchronous BC‐TC and 173 had metachronous BC‐TC. The estimated 5‐year RFS was 91.0% and 96.8%, respectively (*P* = 0.016; Figure S3). Variables with *P* < 0.10 in the univariate Cox regression were then included in the multivariate Cox regression analysis. Synchronous BC‐TC (risk ratio [RR] = 5.597; 95% CI: 1.349‐23.225; *P* = 0.018) and a high tumor grade (RR = 5.476; 95% CI: 1.283‐23.376; *P* = 0.022) were the only two independent prognostic factors for inferior RFS (Figure 2).

## 4 DISCUSSION

Our study demonstrated that patients with BC have a significantly higher risk of being diagnosed with TC, especially micropapillary TC without lymph node metastasis. The incidence of TC was 1.8% in 13 978 patients with BC, and the SIR of secondary TC for female patients with BC was as high as 4.48 (95% CI, 3.95‐5.44) compared with Chinese females in the general population and was 2.52 (95% CI, 2.22‐3.06) compared with areas of high urbanization. These results are comparable with those of previous studies, which showed an SIR ranging from 1.16 to 4.75 in patients with BC.^3^, ^4^ More active surveillance of BC could lead to more incidental diagnoses of TC. In our cohort, as many as 68.5% of patients had microcarcinoma and 67.8% had no lymph node metastasis, indicating early detection of TC in these patients. These results are in agreement with previous studies showing that TC in patients with BC was diagnosed at an earlier stage than ordinary TC and is specific for papillary TC.^5^, ^7^ Similarly, An et al^3^ reported that after excluding incidentally detected cases by imaging modalities during follow‐up of BC, the SIR for secondary TC decreased from 2.18 to 1.73 in a Korean cohort.

Nevertheless, the risk of developing TC after primary BC is higher than the overall risk of developing other SPMs,^6^ and active surveillance is not the only explanation for this phenomenon. An interesting finding of the current study is that a family history of malignancy was the only independent predictor of TC in patients with BC (*P* = 0.025). This result correlates with previous studies showing that patients with BC with other SPMs were more likely to have a first‐degree relative with cancer than were patients without SPMs^8^ and that the incidence of TC was elevated among first‐degree relatives with BC.^9^

These observations suggest that a germ line mutation might play a role in the development of concurrent TC and BC. A well‐known gene mutation‐related disease is Cowden syndrome, which involves splice variants of the tumor suppressor gene PTEN and mutations of CHEK2.^10^, ^11^ Patients with Cowden syndrome have an increased risk of both BC and TC, and the prevalence of Cowden syndrome is approximately 1 in 200 000.^12^ Mutations in the succinate dehydrogenase gene (SDHx) and KLLN were recently reported to cause Cowden syndrome and Cowden‐like syndrome^13^, ^14^ and to thus contribute to the co‐occurrence of BC and TC.^15^

Two previous studies have suggested that the expression of ER and PR was significantly higher in patients with both BC and TC than in patients with BC alone, and ER signaling might be a common etiological factor in TC and BC.^3^, ^4^ However, both of these studies conducted 1:3‐matched groups according to the age at diagnosis and time of surgery, indicating a potential risk of selection bias. In the current study, however, we failed to connect BC and TC co‐occurrence with either ER positivity or luminal subtype in our cohort of 13 978 patients with BC with a known ER/PR status. We also did not identify a connection between the ER/PR status and TC lymph node metastasis, increased tumor size, or multifocality, indicating that the ER status in patients with BC is not associated with TC invasiveness.

External beam radiation therapy has been hypothesized to cause the high co‐occurrence of TC and BC.^16^ However, the typical latency period of TC after radiation exposure is 20 to 30 years, which does not explain the elevated SIR within a short period of time.^17^ Huang et al^18^ concluded that the relative risk of radiation‐associated TC after initial external beam radiation therapy for BC was 1.0 (95% CI, 0.7‐1.5) after a 10‐year follow‐up.

Limited data are available regarding the effect of TC on the survival of patients with BC. In our survival analysis, we reasonably considered that patients in the BC‐TC group had non‐inferior RFS compared with those in the BC‐alone group because of the excellent prognosis of TC itself. Thus, TC was not an independent prognostic factor for RFS in patients with BC. Interestingly, TC was more often identified at an earlier stage in patients with BC, possibly because patients would not undergo surgical treatment of thyroid nodules given the poorer prognosis of advanced BC. However, a worse RFS was identified in patients with synchronous vs metachronous BC‐TC, indicating that these two scenarios might be driven by different mechanisms. While synchronous BC‐TC could be driven by the common germ line mutations mentioned above, metachronous BC‐TC was more likely to be a sporadic event and thus display different biological features.

There are several limitations of our study. First, the study was limited by its retrospective nature. Second, the relatively short follow‐up period prevented us from observing more cancer‐related events and cancer‐specific survival. Finally, although worse survival was identified in patients with synchronous BC‐TC, the underlying mechanism for this is unknown. Because TC is often an indolent tumor that might be undetected for years,^19^ the screening and treatment of TC in patients with BC warrant more evidence to provide benefits to this population.

In conclusion, we identified a family history of malignancy was the only predictor of secondary TC in patients with BC, and an age of >50 years was associated with a larger TC size. Although patients with both BC and TC did not show inferior outcomes, patients with synchronous BC‐TC had poorer RFS than patients with metachronous BC‐TC. Based upon the results of the current study, we propose that there might be different mechanisms behind synchronous and metachronous BC‐TC.

## CONFLICTS OF INTEREST

The authors declare that there is no conflict of interest.
