# Supplementary material for: Association between breast cancer and thyroid cancer: A study based on 13 978 patients with breast cancer
Source: Cancer Med. 2018 Nov 27;7(12):6393–400. doi: 10.1002/cam4.1856 (PMC6308067; doi:10.1002/cam4.1856)
Supplement: Supplementary file 4 [file CAM4-7-6393-s004.docx]

Supplementary Table 1. Clinical-pathological characteristics of BC-TC patients according to lymph node metastasis of thyroid cancer.

| Variables | Total | LN negative | LN positive | *P*-value |
| --- | --- | --- | --- | --- |
| Age (N=202), ≤50 | 115 (56.9%) | 74 (54.0%) | 41 (63.1%) | 0.224 |
| Menopause (N=180), Yes | 99 (55.0%) | 69 (54.8%) | 30 (55.6%) | 0.922 |
| Family history of malignancy (N=198), Yes | 75 (37.9%) | 51 (38.1%) | 24 (37.5%) | 0.939 |
| Pathology of BC (N=202), Invasive | 174 (86.1%) | 119 (86.9%) | 55 (84.6%) | 0.666 |
| Tumor size^†^ (N=184), >2cm | 61 (33.2%) | 42 (32.6%) | 19 (34.5%) | 0.793 |
| Tumor Grade (N=146), III | 37 (25.3%) | 25 (23.1%) | 12 (31.6%) | 0.304 |
| pN (N=202), pN0 | 141 (69.8%) | 96 (70.1%) | 45 (69.2%) | 0.354 |
| LVI (N=136), Yes | 33 (24.3%) | 20 (20.8%) | 13 (32.5%) | 0.148 |
| ER (N=195), Positive | 141 (72.3%) | 98 (72.6%) | 43 (71.7%) | 0.894 |
| PR (N=196), Positive | 125 (63.8%) | 87 (64.0%) | 38 (63.3%) | 0.932 |
| HER2^‡^ (N=183), Positive | 48 (26.2%) | 36 (28.3%) | 12 (21.4%) | 0.327 |
| Ki67 (N=125), ≥20% | 59 (47.2%) | 46 (50.5%) | 13 (38.2%) | 0.220 |
| Subtype^‡^ (N=174) |  |  |  |  |
| Luminal A | 31 (17.8%) | 23 (19.2%) | 8 (14.8%) | 0.250 |
| Luminal B | 94 (54.0%) | 63 (52.5%) | 31 (57.4%) |  |
| HER2-enriched | 23 (13.2%) | 19 (15.8%) | 4 (7.4%) |  |
| Triple-negative | 26 (14.9%) | 15 (12.5%) | 11 (20.4%) |  |

^†^ Size of invasive disease on final pathology

^‡^ Only HER2 status in invasive disease was analyzed

Abbreviations: BC breast cancer; pN pathological N stage; LVI lymph-vascular invasion; ER estrogen receptor; PR progesterone receptor; HER2 human epidermal receptor 2

Supplementary Table 2. Clinical-pathological characteristics of BC-TC patients according to multifocality of thyroid cancer.

| Variables | Total | Multifocal | Unifocal | *P*-value |
| --- | --- | --- | --- | --- |
| Age (N=185), ≤50 | 107 (57.8%) | 32 (60.4%) | 75 (56.8%) | 0.658 |
| Menopause (N=165), Yes | 72 (43.6%) | 20 (42.6%) | 52 (44.1%) | 0.859 |
| Family history of cancer (N=181), Yes | 70 (38.7%) | 23 (44.2%) | 47 (36.4%) | 0.330 |
| Pathology of BC (N=185), Invasive | 160 (86.5%) | 49 (92.5%) | 111 (84.1%) | 0.133 |
| Tumor size^†^ (N=169), >2cm | 56 (33.1%) | 17 (34.0%) | 39 (32.8%) | 0.877 |
| Tumor Grade (N=134), III | 33 (24.6%) | 9 (25.7%) | 24 (24.2%) | 0.862 |
| pN (N=185), pN0 | 126 (68.1%) | 34 (64.2%) | 92 (69.7%) | 0.689 |
| LVI (N=136), Yes | 31 (25.0%) | 11 (28.9%) | 20 (23.3%) | 0.500 |
| ER (N=178), Positive | 130 (73.0%) | 38 (73.1%) | 92 (73.0%) | 0.993 |
| PR (N=179), Positive | 115 (64.2%) | 33 (63.5%) | 82 (64.6%) | 0.889 |
| HER2^‡^ (N=183), Positive | 37 (25.2%) | 11 (25.0%) | 26 (25.2%) | 0.975 |
| Ki67 (N=114), ≥20% | 54 (47.4%) | 14 (38.9%) | 40 (51.3%) | 0.218 |
| Subtype^‡^ (N=140) |  |  |  |  |
| Luminal A | 26 (18.6%) | 10 (21.7%) | 16 (17.0%) | 0.305 |
| Luminal B | 76 (54.3%) | 23 (50.0%) | 53 (56.4%) |  |
| HER2-enriched | 16 (11.4%) | 3 (6.5%) | 13 (13.8%) |  |
| Triple-negative | 22 (15.7%) | 10 (21.7%) | 12 (12.8%) |  |

^†^ Size of invasive disease on final pathology

^‡^ Only HER2 status in invasive disease was analyzed

Abbreviations: BC breast cancer; pN pathological N stage; LVI lymph-vascular invasion; ER estrogen receptor; PR progesterone receptor; HER2 human epidermal receptor 2

Supplementary Table 3. Clinical-pathological characteristics of BC-TC patients according to size of thyroid cancer.

| Variables | Total | <=1cm | >1cm | *P*-value |
| --- | --- | --- | --- | --- |
| Age (N=181), ≤50 | 105 (58.0%) | 79 (63.7%) | 26 (45.6%) | 0.022 |
| Menopause (N=161), Yes | 68 (42.2%) | 40 (36.7%) | 28 (53.8%) | 0.039 |
| Family history of cancer (N=177), Yes | 68 (38.4%) | 46 (38.0%) | 22 (39.3%) | 0.872 |
| Pathology of BC (N=181), Invasive | 157 (86.7%) | 109 (87.9%) | 48 (84.2%) | 0.496 |
| Tumor size^†^ (N=165), >2cm | 54 (32.7%) | 38 (33.6%) | 16 (30.8%) | 0.716 |
| Tumor Grade (N=131), III | 32 (24.4%) | 20 (20.8%) | 12 (34.3%) | 0.113 |
| pN (N=181), pN0 | 122 (67.4%) | 80 (64.5%) | 42 (73.7%) | 0.461 |
| LVI (N=121), Yes | 32 (26.4%) | 25 (29.4%) | 7 (19.4%) | 0.256 |
| ER (N=174), Positive | 127 (73.0%) | 92 (76.0%) | 35 (66.0%) | 0.172 |
| PR (N=175), Positive | 175 (64.0%) | 82 (67.2%) | 30 (56.6%) | 0.179 |
| HER2^‡^ (N=163), Positive | 42 (25.8%) | 31 (27.4%) | 11 (22.0%) | 0.464 |
| Ki67 (N=111), ≥20% | 53 (47.7%) | 38 (48.1%) | 15 (46.9%) | 0.907 |
| Subtype^‡^ (N=155) |  |  |  |  |
| Luminal A | 25 (16.1%) | 18 (16.5%) | 7 (15.2%) | 0.498 |
| Luminal B | 89 (57.4%) | 66 (60.6%) | 23 (50.0%) |  |
| HER2-enriched | 18 (11.6%) | 11 (10.1%) | 7 (15.2%) |  |
| Triple-negative | 23 (14.8%) | 14 (12.8%) | 9 (19.6%) |  |

^†^ Size of invasive disease on final pathology

^‡^ Only HER2 status in invasive disease was analyzed

Abbreviations: BC breast cancer; pN pathological N stage; LVI lymph-vascular invasion; ER estrogen receptor; PR progesterone receptor; HER2 human epidermal receptor 2

Supplementary Table 4. Clinical-pathological characteristics of BC-TC patients according to the time of diagnosis of TC.

| Variables | Total | T-B | T=B | B-T | *P*-value |
| --- | --- | --- | --- | --- | --- |
| Age (N=247), ≤50 | 137 (55.5%) | 19 (44.2%) | 38 (51.4%) | 80 (61.5%) | 0.097 |
| Menopause (N=224), Yes | 104 (46.4%) | 24 (55.8%) | 29 (42.6%) | 51 (45.1%) | 0.370 |
| Family history of cancer (N=242), Yes | 89 (36.8%) | 17 (39.5%) | 25 (35.2%) | 47 (36.7%) | 0.898 |
| Pathology of BC (N=247), Invasive | 209 (84.6%) | 33 (76.7%) | 63 (85.1%) | 113 (86.9%) | 0.273 |
| Tumor size^†^ (N=165), >2cm | 73 (38.6%) | 11 (35.5%) | 22 (39.3%) | 40 (39.2%) | 0.926 |
| Tumor Grade (N=176), III | 47 (26.7%) | 8 (26.7%) | 18 (32.7%) | 21 (23.1%) | 0.442 |
| pN (N=181), pN0 | 169 (68.4%) | 33 (76.7%) | 51 (68.9%) | 85 (65.4%) | 0.545 |
| LVI (N=171), Yes | 44 (25.7%) | 5 (17.2%) | 19 (33.9%) | 20 (23.3%) | 0.188 |
| ER (N=239), Positive | 174 (72.8%) | 32 (74.4%) | 55 (77.5%) | 87 (69.6%) | 0.476 |
| PR (N=240), Positive | 161 (67.1%) | 31 (72.1%) | 52 (73.2%) | 78 (61.9%) | 0.198 |
| HER2^‡^ (N=191), Positive | 48 (25.1%) | 6 (18.2%) | 17 (30.4%) | 25 (24.5%) | 0.432 |
| Ki67 (N=157), ≥20% | 78 (49.7%) | 13 (43.3%) | 29 (52.7%) | 36 (50.0%) | 0.708 |
| Subtype^‡^ (N=208) |  |  |  |  |  |
| Luminal A | 41 (19.7%) | 11 (29.7%) | 14 (21.9%) | 16 (15.0%) | 0.507 |
| Luminal B | 111 (53.4%) | 16 (43.2%) | 35 (54.7%) | 60 (56.1%) |  |
| HER2-enriched | 27 (13.0%) | 6 (16.2%) | 7 (10.9%) | 14 (13.1%) |  |
| Triple-negative | 29 (13.9%) | 4 (10.8%) | 8 (12.5%) | 17 (15.9%) |  |

^†^Size of invasive disease on final pathology

^‡^ Only HER2 status in invasive disease was analyzed

Abbreviations: BC breast cancer; pN pathological N stage; LVI lymph-vascular invasion; ER estrogen receptor; PR progesterone receptor; HER2 human epidermal receptor 2

Supplementary Table 5. Univariate Logistic regression analysis of thyroid cancer in breast cancer patients.

| Variants | Exposure | 95% CI | *P* value |
| --- | --- | --- | --- |
| Age >50 vs. <=50 | 0.786 | 0.611-1.013 | 0.063 |
| Menopause Yes vs. No | 0.717 | 0.550-0.934 | 0.014 |
| ER negative vs. positive | 0.965 | 0.724-1.287 | 0.810 |
| PR negative vs. positive | 0.978 | 0.745-1.283 | 0.871 |
| Grade III vs. I/II | 1.325 | 0.946-1.855 | 0.102 |
| Family history of malignancy Yes vs. No | 1.520 | 1.167-1.979 | 0.002 |
| pT2-4 vs. pTis-1 | 0.686 | 0.518-0.909 | 0.009 |
| pN1 vs. pN0 | 0.854 | 0.620-1.177 | 0.335 |
| pN2-3 vs. pN0 | 0.686 | 0.461-1.019 | 0.062 |
| LVI Yes vs. No | 0.718 | 0.508-1.014 | 0.060 |
| Ki67 ≥20% vs. <20% | 0.804 | 0.586-1.103 | 0.176 |
| HER2 Positive vs. negative | 1.039 | 0.771-1.400 | 0.802 |
| Luminal B vs. Luminal A | 0.673 | 0.469-0.967 | 0.032 |
| HER2-enriched vs. Luminal A | 0.846 | 0.518-1.383 | 0.506 |
| Triple-negative vs. Luminal A | 0.746 | 0.461-1.206 | 0.231 |

Abbreviations: CI confidence interval; pT pathological T stage; pN pathological N stage; LVI lymph-vascular invasion; ER estrogen receptor; PR progesterone receptor; HER2 human epidermal receptor 2
